# Evaluation of the effects of different irrigation activation techniques on the surface roughness of dentin and blood clot in regenerative endodontic models

**DOI:** 10.1007/s10103-026-04884-7

**Published:** 2026-05-11

**Authors:** Sevgi Bulak Yeliz, Zeliha Uğur Aydın

**Affiliations:** 1 Gülhane Faculty of Dentistry, Department of Endodontics, Sağlık Bilimleri Üniversitesi, Ankara, Turkey; 2 Henry M. Goldman School of Dental Medicine, Department of Endodontics, Boston University, Boston, USA

**Keywords:** Blood clot, Dentin surface roughness, EDTA activation, Regenerative endodontics

## Abstract

Regenerative endodontic treatment (RET) is a biologically based approach aiming to restore the pulp–dentin complex in immature permanent teeth with necrotic pulps. In this procedure, a blood clot is induced within the canal to serve as a scaffold supporting stem cell migration, and its stability and regenerative success are closely linked to dentin surface morphology. This study focused exclusively on the quantitative assessment of surface roughness, without evaluating biological outcomes such as cellular response or long-term clot stability, and aimed to evaluate the effects of 17% EDTA activated by conventional needle irrigation (CNI), passive ultrasonic irrigation (PUI), EDDY, and SWEEPS on dentin and blood clot surface roughness using widefield confocal microscopy (CM). Seventy-five single-rooted maxillary incisors were decoronated and standardized to 11 ± 1 mm root length. Following instrumentation with VDW Reciproc R40 files and apical enlargement to 1.3 mm using Gates Glidden drills, canals received 20 mL of 17% EDTA for 5 min. In the activated groups, 1.5 mL EDTA was activated for 1 min, whereas the control group was irrigated with 20 mL saline. Specimens were split, and Sa values were measured on dentin and post-blood-clot surfaces using widefield CM. No significant differences were observed between canal regions for any technique regarding dentin and clot surface roughness (*p* > .05). SWEEPS and EDDY produced higher Sa values on dentin surfaces than CNI in middle and apical regions (*p* < .05). After blood application, the control group showed significantly higher Sa values, especially apically (*p* < .05). Activation of EDTA using PUI, EDDY, or SWEEPS enhanced dentin surface roughness, which may influence clot adhesion and stability in RET.

## Introduction

Regenerative endodontic treatments (RETs) are cell-based contemporary approaches aimed at restoring the biological functions of the dentin–pulp complex [1]. The success of this treatment process depends on the biological conditioning of the root canal dentin surface. The chemical preparation of the canal surface plays a critical role in promoting the adhesion, proliferation, and differentiation of mesenchymal stem cells into odontoblast-like cells along the canal walls [2]. In this context, 17% ethylenediaminetetraacetic acid (EDTA), one of the most used irrigation agents, facilitates the removal of the smear layer by partially demineralizing dentin, thereby exposing dentinal tubules and collagen fibrils. Moreover, by modifying the dentin surface, EDTA enhances stem cell attachment and supports cellular interactions [3–5].

Biologically active molecules such as Transforming Growth Factor Beta 1 (TGF-β1), Vascular Endothelial Growth Factor (VEGF), and Fibroblast Growth Factor-2 (FGF-2), released from the dentin surface treated with EDTA, play a critical role in directing key regenerative processes such as cell proliferation, angiogenesis, and tissue differentiation [2, 6]. Additionally, the blood clot induced into the canal in RET protocols serves as a natural biological scaffold for stem cell migration and organization, and the dentin surface must be properly prepared to transmit biological signals in order to maintain the effectiveness of this scaffold [4].

In RET, the blood clot functions as a natural scaffold that facilitates the adhesion, migration, and proliferation of mesenchymal stem cells. Among the key structural properties of biological scaffolds, porosity plays a critical role by modulating cellular infiltration, nutrient exchange, and subsequent tissue regeneration [7, 8]. Although the biological significance of blood clot formation has been widely studied, the potential impact of different EDTA activation methods on its physical characteristics remains largely unexplored. Given EDTA’s known anticoagulant effects, variations in its activation may influence clot formation dynamics. Accordingly, the surface roughness of the blood clot may serve as a surrogate parameter reflecting scaffold-level alterations, particularly related to its microstructural organization and inferred porosity.

However, some studies have reported that the blood clotting process within the root canal systems may be adversely affected by the anticoagulant effect of EDTA [9, 10]. This has been attributed to the disruption of the coagulation cascade due to the chelation of calcium ions—an essential component of clot formation—by EDTA [11]. Taweewattanapaisan et al. [9], demonstrated, through Scanning Electron Microscopy (SEM) imaging, a decrease in the density of the fibrin network and alterations in erythrocyte morphology within blood clots formed on EDTA-treated dentin surfaces. Vieira et al. [10] reported that the application of EDTA using different final irrigation activation techniques did not result in a significant change in these effects. However, in both studies, only the blood clot was evaluated, and the relationship between dentin surface topography and clot structure was not quantitatively analysed.

On the other hand, some studies investigating the morphological changes on the dentin surface following final irrigation activation [12, 13] have generally utilized advanced imaging techniques such as atomic force microscopy (AFM).These studies have demonstrated that irrigation duration, irrigant concentration, and activation methods can lead to significant changes in dentin surface roughness. However, no study in the literature has simultaneously and quantitatively evaluated both the surface morphology of the dentin and the blood clot formed on it.

Different activation methods employed in irrigation protocols have been developed to enhance the efficacy of irrigants. CNI is the basic method that passively delivers the irrigant into the root canal; however, it may limit effective irrigant circulation in the apical region [14]. PUI improves the flow dynamics of the irrigant using ultrasonic vibrations and enhances irrigation efficacy by transmitting mechanical energy to the dentin surface [15]. The continuous vibration of the ultrasonic file generates stable acoustic streaming and cavitation, improving cleaning efficiency even in areas not directly touched by the instrument, and has been shown to significantly increase dentin surface roughness [16]. The EDDY system activates the irrigant at a high frequency (6,000 Hz) using polymer tips that provide sonic activation, ensuring effective irrigation with minimal damage to root dentin [17]. Its flexible polyamide tip produces three-dimensional oscillation within the canal, which enhances irrigant replacement and smear layer removal, while preserving dentin structure [18]. The Shock Wave Enhanced Emission Photoacoustic Streaming (SWEEPS) technology employs Erbium-doped Yttrium Aluminum Garnet (Er: YAG) laser energy with dual laser pulses to generate rapidly expanding and collapsing cavitation bubbles within the irrigant, thereby enhancing the penetration and effectiveness of the irrigant within the root canal system [19, 20].

The aim of this study was to quantitatively evaluate, using widefield CM, how different EDTA activation techniques (CNI, PUI, EDDY, and SWEEPS) affect the surface roughness of both dentin and the formed blood clot following the application of 17% EDTA, in order to better understand their potential implications for scaffold integration and regenerative outcomes. The null hypothesis of the study was that different final irrigation activation methods would not cause a significant difference in the surface roughness of dentin and the blood clot.

## Materials and methods

### Ethical approval and sample selection

The necessary ethical approval for this study was obtained from the Ethics Committee for Scientific Research of the University of Health Sciences Gülhane (Decision No: 2025 − 101, Date: 11.02.2025). The sample size was calculated based on a power analysis performed using G*Power software version 3.1.2 (Universitat, Düsseldorf, Germany), with reference to a recent study in the literature with a similar design [21], assuming an alpha error probability of 0.05, a power of 80%, and an effect size of 0.25. Based on the power analysis, 15 specimens were required per group, including the control group, totalling 75 samples. Therefore, 75 maxillary incisor teeth extracted for periodontal reasons from patients aged between 18 and 50 years were included, provided they met the eligibility criteria for the experimental design. Teeth with a history of previous endodontic treatment, cracks, internal or external resorption, calcified canal structures, or immature apices were excluded from the study. All root canal procedures and surface roughness analyses were performed by a single operator, an endodontist with 3 years of experience. Following extraction, soft tissue remnants were gently removed from the root surfaces using manual curettes under visual inspection to minimize the risk of crack formation. The teeth were subsequently stored in 0.1% thymol solution at 4 °C for a maximum period of 3 months before use to prevent microbial contamination while preserving the structural integrity of dentin.

### Root canal preparation and open apex simulation

Conventional endodontic access cavities were prepared in all teeth under water cooling using an air turbine and diamond burs (G&Z Instrumente, Lustenau, Austria). Following access cavity preparation, apical patency was verified using a #10 K-type hand file (Perfect, Shenzhen, China). The teeth were decoronated 2 mm apical to the cementoenamel junction using a diamond disc (Sunshine Diamonds, Langenhagen, Germany), and the roots were standardized to a length of approximately 11 ± 1 mm by performing a second cut 11 mm apical to the initial cutting point.

Root canals were shaped using a torque-controlled endomotor operating in reciprocation mode (Woodpecker AI Endomotor, Guilin, China) and Reciproc #R40 file (VDW, Munich, Germany). During preparation, irrigation was performed with 5 mL of 1.5% NaOCl (Speiko, Münster, Germany) and 20 mL of saline solution using a 30 G side-vented needle (Endo-Top, Cerkamed, Stalowa Wola, Poland). To simulate an immature apex, the apical foramen was enlarged to a diameter of 1.3 mm under water cooling using Gates-Glidden burs #1–5 (Proud, London, UK), as referenced in a similar study [10]. Longitudinal grooves were then created on the buccal and palatal surfaces of the roots using a diamond disc (Sunshine Diamonds), ensuring that the canal lumen was not perforated.

To create a reservoir for the irrigant during irrigation, an artificial pulp chamber was prepared in the coronal portion of the canal (4 ± 1 mm) using diamond burs (Sunshine Diamonds), in reference to a similar study [22]. The coronal, middle, and apical regions were defined by drawing two parallel horizontal lines at 2 mm intervals on the root surface. The specimens were then embedded in silicone impression material (Coltene/Whaledent, Langenau, Germany) to simulate periodontal tissue resistance and create a closed irrigation system, in accordance with previously published in vitro models [23].

### EDTA activation protocols

The specimens were randomly assigned to five groups (Control, CNI, PUI, EDDY, and SWEEPS), and the final irrigation activation protocols were carried out in accordance with the procedures outlined by Vieira et al. [10].

#### Control

In this group, 20 mL of saline solution (pH 6) at room temperature was delivered into the root canal using a 30 G side-vented needle (Endo-Top, Cerkamed, Stalowa Wola, Poland) positioned 2 mm short of the working length, at a flow rate of 20 mL over 5 min. During irrigation, the solution was simultaneously aspirated using a suction (Monoart, Euronda, Vicenza, Italy) placed in the coronal access and connected to a vacuum aspiration pump.

#### CNI

In this group, 20 mL of 17% EDTA (pH 7) (Speiko) at room temperature was applied using a 30 G side-vented needle following the protocol described for the control group. This was followed by a final irrigation with 20 mL of saline solution.

#### PUI

In this group, 18.5 mL of the 20 mL 17% EDTA (Speiko) solution was applied following the CNI protocol. The remaining 1.5 mL EDTA was activated in three activation cycles. In each activation cycle, 0.5 mL of EDTA was delivered into the canal for 10 s. An ultrasonic tip (T1; Guilin Woodpecker Medical Instrument Co., Ltd., Guilin, China) connected to the dental unit (Stern Weber S300; Stern Weber, Imola, Italy) set to 50% power in endo mode was inserted into the root canal 2 mm short of the working length and moved in a corono-apical direction with a 4–5 mm amplitude to activate 0.5 mL of EDTA solution (Microvem, Istanbul, Turkey) for 20 s. This procedure was repeated three times, providing a total of 1 min of solution activation within the root canal. Final irrigation was then performed with 20 mL of saline solution.

#### EDDY

In this group, 18.5 mL of the 20 mL 17% EDTA (Speiko) solution was applied following the CNI protocol. The remaining 1.5 mL EDTA was used in three activation cycles. In each activation cycle, 0.5 mL of EDTA was delivered into the canal for 10 s and activated using an EDDY tip (VDW, Munich, Germany) attached to the Sonicmax sonic device (Maximum Dental Inc., Secaucus, NJ, USA) operating at a frequency of 6000 Hz. The tip was inserted 2 mm short of the working length and moved in a corono-apical direction with a 4–5 mm amplitude for 20 s. This procedure was repeated three times, providing a total of 1 min of solution activation within the root canal. Final irrigation was then performed with 20 mL of saline solution.

#### SWEEPS

SWEEPS: In this group, 18.5 mL of the 20 mL 17% EDTA (Speiko) solution was applied following the CNI protocol. The remaining 1.5 mL EDTA was used in three activation cycles. In each activation cycle, 0.5 mL of EDTA was delivered into the canal for 10 s and activated for 20 s using an Er: YAG laser system (LightWalker^®^ AT S, Fotona, Ljubljana, Slovenia) operating in SWEEPS mode (2940 nm, 20 mJ per pulse, 15 Hz, pulse duration 50 µs, output power 0.30 W). A SWEEPS radial fiber tip (600/9) was positioned in the pulp chamber with the water and air spray turned off, as described in previous studies [24, 25]. This procedure was repeated in three cycles (totalling 1 min), followed by final irrigation with 20 mL of saline solution.

### Specimen sectioning and initial surface roughness measurement

After completion of the final irrigation activation protocols and drying of the root canals with sterile paper points, the specimens were carefully split into two halves along the previously prepared grooves using a scalpel. The half with better structural integrity was selected for evaluation.

Before blood application, the surface roughness of the selected specimen half was evaluated using the Zeiss SmartProof 5 (Carl Zeiss, Germany) widefield CM. Surface roughness measurements were performed from three distinct areas corresponding to the coronal, middle, and apical regions, each with a size of 0.2 × 0.2 mm², which was selected based on previous literature to ensure repeatability and adequate resolution of dentin surface topography [26]. Image analysis and surface roughness measurements were performed by a single operator blinded to the group allocation. To assess intra-operator reliability, 20% of the samples were re-evaluated after a two-week interval, and the intraclass correlation coefficient (ICC) was calculated (ICC = 0.92), indicating excellent reliability. For measurement standardization, surface roughness measurements from each canal third were performed in triplicate, and the mean values were used for statistical analysis.

### Blood application and second surface roughness measurement

The blood sample was obtained from a healthy female volunteer with no systemic disease, no history of smoking, and no use of anticoagulant medication, in accordance with the World Health Organization’s [27] recommended finger-prick blood collection protocol. After discarding the first drop with a sterile gauze pad, 5 µL of blood from the second drop was carefully placed onto the previously measured dentin surface. The specimens were incubated at 37 °C for 10 min to allow clot formation. At the end of the incubation period, surface roughness measurements were repeated from the coronal, middle, and apical regions to evaluate the surface roughness of the blood clot.

### Topographic evaluation and roughness analysis

**Fig. 1:**
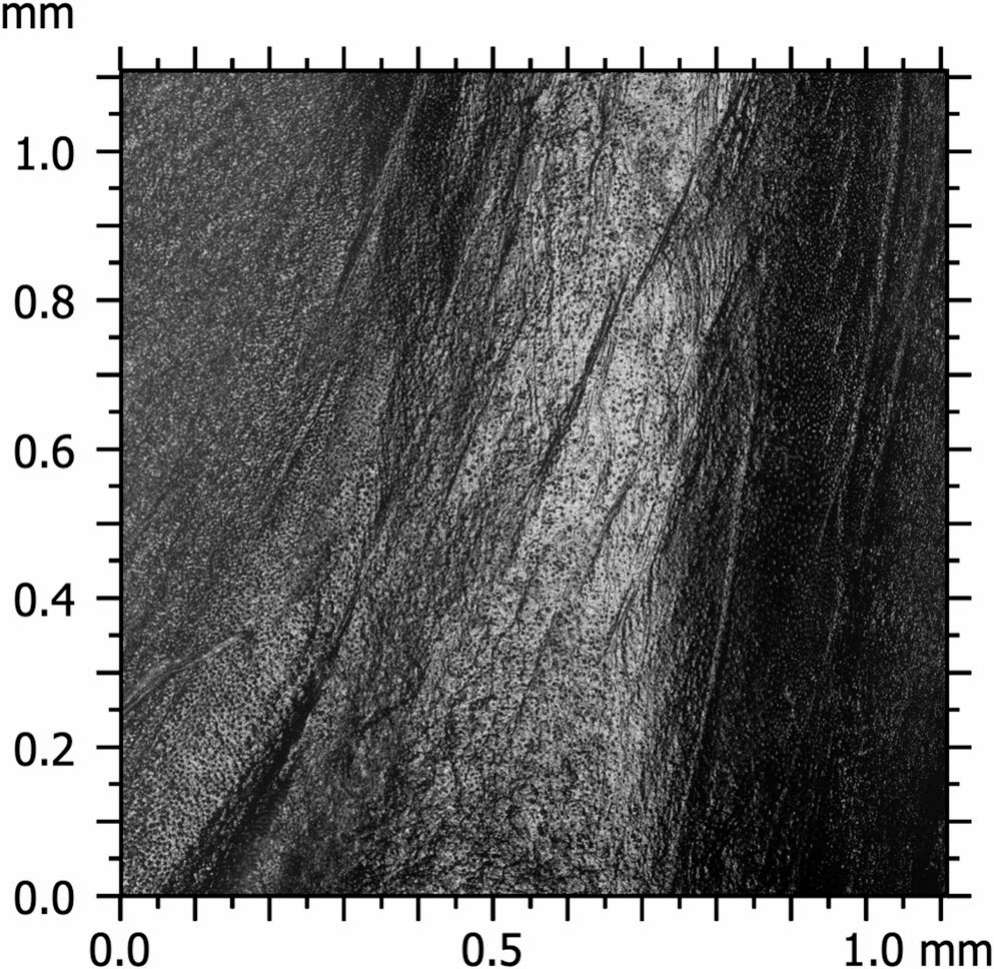
Obtaining the black-and-white micrograph of the surface

**Fig. 2:**
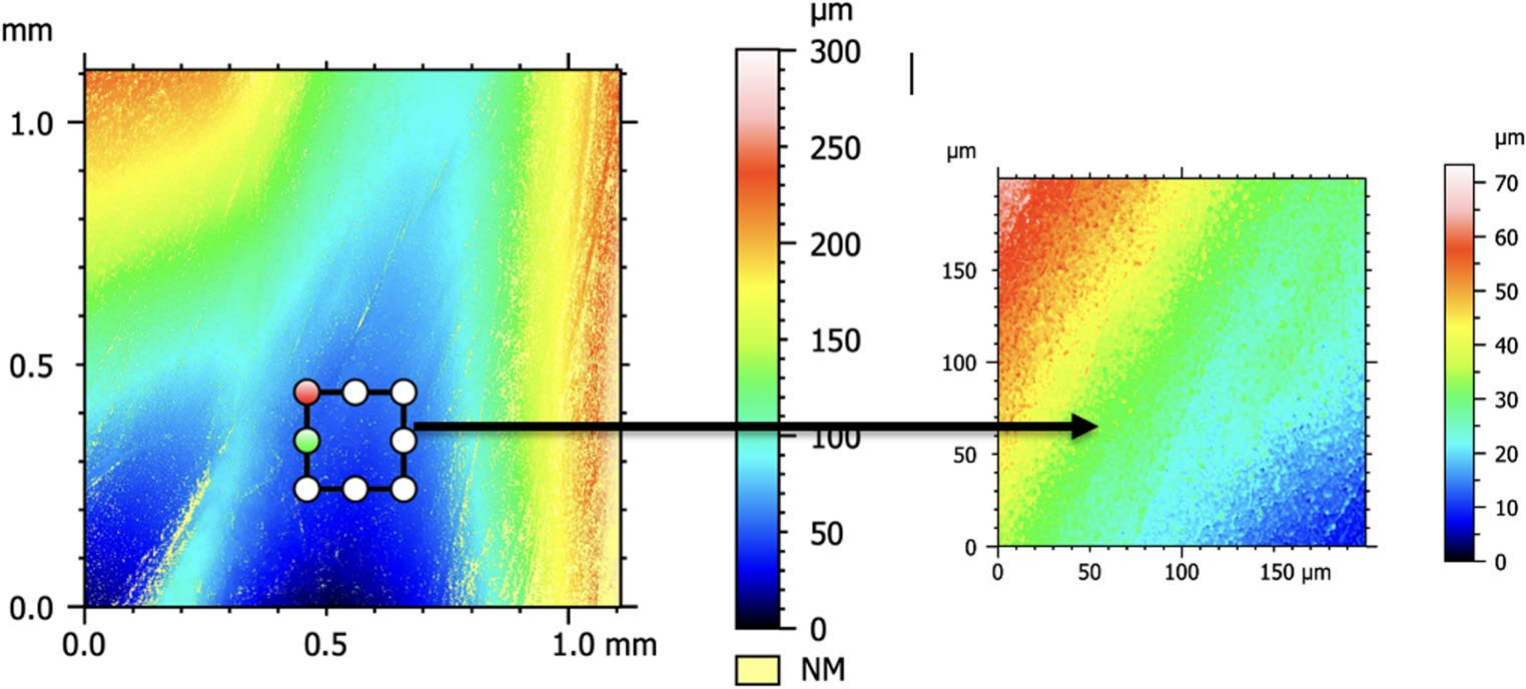
Creation of a color-coded height map from the images and selection of a representative area measuring 0.2 mm × 0.2 mm from this map

**Fig. 3:**
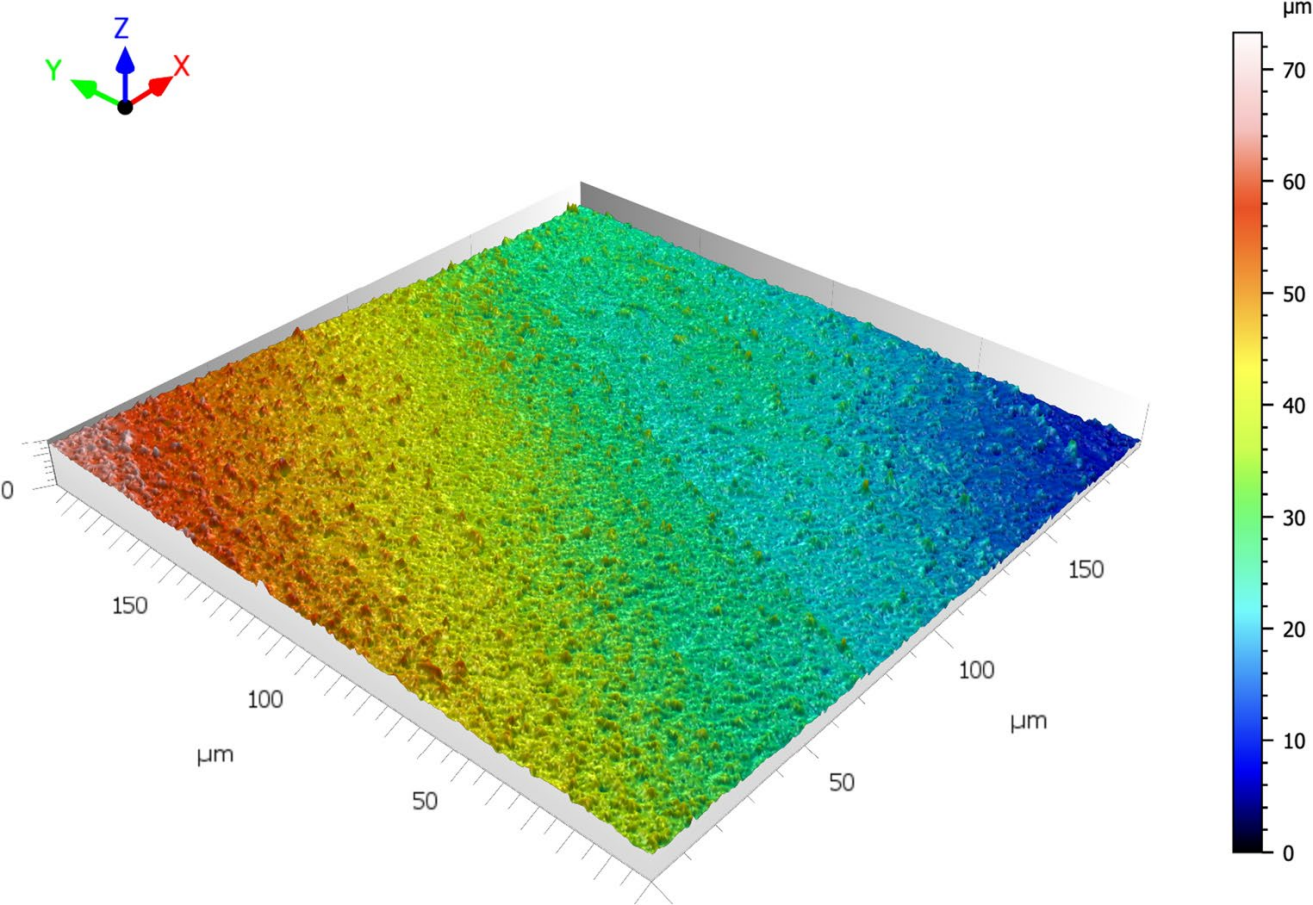
Generation of a detailed 3D topographic map of the surface from the selected area

The acquired images were analysed using ConfoMap ST 9.3.10494 software (Zeiss, Germany). First, black-and-white micrographs were recorded to evaluate the raw surface morphology of the specimens and to observe the overall structure of the surface topography (Fig. 1). Subsequently, a color-coded height map was generated to perform a quantitative roughness analysis of the surface, and from this map, a representative area measuring 0.2 mm × 0.2 mm was selected to reflect the surface characteristics (Fig. 2). This area was positioned to best represent the micro-topographic variations on the surface and to assess the height differences in various regions. Detailed 3D topographic maps of the selected areas were then created (Fig. 3). As a result of this analysis, key roughness parameters such as Sa (arithmetical mean height), Sq (root mean square height), Sp (maximum peak height), Sv (maximum valley depth), and Sz (peak-to-valley height) were obtained in micrometres (µm). A study evaluating root canal roughness [26] was used as a reference, and the Sa values—which express the average height differences within a defined area in three-dimensional surface measurements—were recorded from the coronal, middle, and apical regions of each root half for statistical analysis.

### Statistical analysis

All statistical analyses were performed using IBM SPSS Statistics for Windows, Version 25.0 (IBM Corp., Armonk, NY, USA). The distribution of the data was assessed using the Shapiro-Wilk test. Homogeneity of variances was evaluated using Levene’s test prior to performing ANOVA analyses, and the assumption was confirmed for the relevant comparisons. One-way analysis of variance (One-Way ANOVA) and post hoc Tukey tests were used to compare the Sa values obtained after irrigation and after blood application across all tested final irrigation activation methods. The same statistical tests were also applied to compare the Sa values obtained from the apical, middle, and coronal regions within the same activation method, as well as to compare different activation methods within the same root region. Paired sample t-tests were used to compare the Sa values obtained from the same region before and after blood application. In addition, to control for the potential inflation of Type I error due to multiple analytical sets across different canal sections and time points, Bonferroni-adjusted significance thresholds were applied where appropriate. The level of significance was set at *p* < .05.

## Results

No statistically significant differences were observed among the regions in terms of dentin Sa values or post-blood application clot surface Sa values for any of the tested activation techniques (*p* > .05) (Tables 1 and 2).

**Table 1:** After Irrigation Procedures and Before Blood Application, Dentin Surface Roughness Values (mean ± standard deviation) (Sa, µm)

	CONTROL	CNI	PUI	EDDY	SWEEPS
CORONAL	6.23 ± 1.78^ax^	8.62 ± 1.15^ay^	10.76 ± 1.27^az^	11.34 ± 2.13^az^	11.07 ± 2.09^az^
MIDDLE	6.47 ± 1.08^ax^	9.44 ± 1.01^ay^	10.54 ± 1.13^ayz^	10.67 ± 1.55^az^	11.02 ± 1.10^az^
APICAL	5.25 ± 1.97^ax^	9.23 ± 1.36^ay^	11.66 ± 1.41^az^	11.22 ± 2.11^az^	9.97 ± 1.44^ayz^

*Differences between columns are indicated by a, b, c; differences between rows are indicated by x, y, z

**Table 2:** After Blood Application, Blood Surface Roughness Values (mean ± standard deviation) (Sa, µm)

	CONTROL	CNI	PUI	EDDY	SWEEPS
CORONAL	10.48 ± 1.40^ax^	7.98 ± 2.30^ay^	7.81 ± 1.18^az^	8.28 ± 1.51^az^	7.84 ± 1.41^az^
MIDDLE	9.72 ± 1.53^ax^	8.65 ± 1.53^ay^	8.84 ± 1.69^ay^	8.72 ± 1.29^ay^	8.10 ± 1.05^ay^
APICAL	9.85 ± 1.35^ax^	8.61 ± 1.79^ay^	9.51 ± 2.47^az^	9.10 ± 1.99^ayz^	8.77 ± 1.43^ayz^

*Differences between columns are indicated by a, b, c; differences between rows are indicated by x, y, z

Following irrigation, the lowest Sa value in the coronal region was measured in the control group, followed by the CNI group. No statistically significant differences were found among the Sa values of PUI, EDDY, and SWEEPS in the coronal region (*p* > .05). In the middle region the control group exhibited the lowest Sa values, while SWEEPS and EDDY showed significantly higher Sa values compared to CNI (*p* < .05). No significant difference was observed between CNI and PUI in the middle region (*p* > .05). In the apical region, the control group again demonstrated the lowest Sa value; although there was no significant difference between SWEEPS and CNI (*p* > .05), the Sa values of PUI and EDDY were significantly higher than those of CNI (*p* < .05) (Table 1).

Following blood clot application, the control group exhibited higher Sa values in the coronal region compared to the other groups, while no statistically significant differences were observed among the other activation methods (*p* > .05). Similarly, in the middle region, the control group demonstrated significantly higher Sa values than all other activation techniques, with no significant differences detected among the activation methods themselves (*p* > .05). In the apical region, the control group again showed significantly higher Sa values compared to the other activation techniques (*p* < .05). Additionally, a statistically significant difference was noted between PUI and CNI in the apical region (*p* < .05) (Table 2).

When comparing Sa values before irrigation and after blood clot application, an increase in Sa values was observed in the control group in the coronal region following blood application, whereas PUI, EDDY, and SWEEPS demonstrated a significant decrease (*p* < .05). In the CNI group, no statistically significant difference was found between pre- and post-blood application Sa values in the coronal region (*p* > .05). In both the middle and apical regions, Sa values increased in the control group following blood application, whereas a significant decrease was observed in the other irrigation activation techniques (*p* < .05). No significant difference was found between pre- and post-blood application Sa values in the CNI group in the apical region (*p* > .05) (Tables 1 and 2).

## Discussion

This study analysed the effects of different EDTA activation techniques on root canal dentin surface morphology and the topographical characteristics of the subsequently formed blood clot in immature tooth models using widefield CM. Based on the findings obtained, the null hypothesis was rejected, as statistically significant differences were observed in dentin and clot surface roughness values depending on the activation technique used for 17% EDTA irrigation followed by blood application.

In this study, widefield CM enabled the scanning of broader surface areas compared to conventional SEM, thereby providing data that represent a larger portion of the tooth structure. Although SEM micrographs offer high-magnification detail, they are limited to a relatively small surface area and, therefore, the findings may not always be representative of the entire surface [28]. Moreover, to the best of our knowledge, this is the first study in the literature to quantitatively evaluate the surface topography of the blood clot formed within the root canal.

In all tested irrigation activation protocols, EDTA was applied to the root canal and subsequently rinsed with saline, using standardized procedures to ensure that the solution type, concentration, and application parameters were consistent across all groups. Through this approach, all variables other than the final irrigation activation techniques were controlled. Following each activation method, no statistically significant differences in Sa values were observed among the coronal, middle, and apical regions of the dentin surface. Due to the limited number of studies directly evaluating this parameter in the literature, our results were interpreted indirectly. In contrast to the findings of the present study, Elsheikh et al. [29] using AFM, reported a significant decrease in surface roughness from the coronal to apical regions following 17% EDTA application with CNI. Similarly, Brînză et al. [30] found higher roughness values in the coronal region and lower values in the apical region after CNI, based on AFM analysis. Variations among findings may be attributed to methodological differences, such as the use of mature teeth or discrepancies in the irrigation activation protocols. In line with our results, Er Karaoğlu et al. [31] reported, via CLSM, that there were no significant differences in tubular penetration across different canal levels following CNI in oval canals filled with Ca(OH)₂. Mancini et al. [24] using field emission scanning electron microscopy (FESEM), observed a progressively increasing smear layer removal efficacy from apical to coronal regions with CNI, sonic, and ultrasonic activation in mandibular premolars, whereas no significant difference among canal regions was noted following SWEEPS activation. Similarly, Uslu et al. [32], employing transmission electron microscopy (TEM), reported an increasing smear removal efficacy from apical to coronal regions with CNI in distal canals of mandibular molars; however, no significant differences were found among canal levels following PUI and SWEEPS activation. It may be hypothesized that the enlarged canal anatomy mimicking immature root morphology allowed for more homogeneous irrigant distribution throughout the canal, resulting in similar EDTA efficacy and thereby the absence of significant differences in surface roughness among the regions across all final irrigation activation techniques.

No significant differences were observed in the Sa values of the blood clot formed in the coronal, middle, and apical regions of any of the specimens in which blood was applied. Consistent with the findings of the present study, Vieira et al. [10], reported that activation of EDTA using different techniques, followed by ultrastructural evaluation of the blood clot with TEM, revealed no significant differences in fibrin network density among canal levels. Similarly, Taweewattanapaisan et al. [9], using SEM after standard needle irrigation with EDTA, observed comparable fibrin network densities across all root canal regions. It may be suggested that since EDTA demonstrated similar surface roughness effects across different canal regions, the blood clots formed in these regions exhibited similar structural characteristics as well.

The blood clot generated in regenerative endodontic procedures not only serves as a biological matrix but also functions as a transient scaffold structure. Its physical characteristics, particularly porosity, are considered important regulators of stem cell migration, proliferation, and regenerative outcomes [7, 8]. In the present study, surface roughness measurements were used as an indirect indicator to evaluate whether different EDTA activation protocols could influence the microstructural characteristics of the clot. Given the anticoagulant nature of EDTA, variations in activation dynamics may influence fibrin organization and scaffold architecture. Although clot porosity was not directly quantified, the differences observed in surface roughness values may reflect subtle alterations in clot morphology, suggesting that irrigation dynamics may influence scaffold formation within the regenerative environment.

No significant differences were observed in Sa values among the coronal, middle, and apical regions of the root canal in teeth where EDTA was activated using PUI, EDDY, or SWEEPS. In agreement with the present findings, Tekinarslan et al. [33], reported, through SEM analysis, that sonically, ultrasonically, and laser-activated EDTA demonstrated comparable efficacy in smear layer removal following post space preparation in single-rooted mandibular teeth. Similarly, Er Karaoğlu et al. [31], using CLSM, found no significant differences in irrigant penetration among the apical, middle, and coronal regions following standard needle irrigation in Ca(OH)₂-treated oval canals. In contrast to our results, Tong et al. [34], reported that in curved root canals, PIPS and SWEEPS were more effective in smear layer removal compared to ultrasonic and sonic activation. All final irrigation activation techniques used in the present study aimed to enhance the dynamic movement of the irrigant. By standardizing the concentration, volume, and delivery rate of EDTA, comparable outcomes were achieved across all final irrigation activation methods.

In the CNI group, Sa values in both the coronal and apical regions were lower compared to those observed with EDDY, PUI, and SWEEPS. Consistent with our findings, Tsenova-Ilieva et al. [12] using AFM, reported that PUI resulted in greater increases in dentin surface roughness than CNI in single-rooted incisors. Similarly, Mancini et al. [24], through FESEM, demonstrated that at 3, 5, and 8 mm from the apex in mandibular premolars, SWEEPS, PUI, and EndoActivator (EA) were more effective than CNI in removing the smear layer. In contrast, Almutairi et al. [25], utilizing TEM, reported that CNI was less effective in smear layer removal compared to PUI and SWEEPS in mandibular premolars. It may be suggested that activation methods enhance EDTA’s penetration into dentinal tubules and increase its inorganic tissue-dissolving capacity through the mechanical energy generated during activation, which may result in higher surface roughness values on dentin.

EDTA activation may exert contrasting effects on dentin and blood clot surfaces. On dentin, the chelating action of EDTA removes the smear layer and induces superficial demineralization, increasing surface roughness and potentially enhancing scaffold retention and stem cell adhesion by exposing collagen fibrils and dentinal tubules [35]. In contrast, the anticoagulant properties of EDTA may interfere with fibrin polymerization, potentially resulting in smoother and less porous clot structures. Therefore, the effects of irrigation activation should be interpreted in a substrate-specific manner rather than as universally beneficial. While increased dentin roughness may facilitate scaffold retention, reduced clot roughness may indicate alterations in clot architecture that could influence cellular infiltration and scaffold stability. These divergent outcomes highlight the importance of evaluating irrigation activation strategies according to their distinct effects on both mineralized dentin surfaces and the biological scaffold formed during regenerative procedures. It should also be emphasized that the relationship between increased dentin surface roughness and improved blood clot stability remains a biologically plausible hypothesis rather than an experimentally confirmed mechanism.

Surface roughness was evaluated using multiple areal parameters (Sa, Sq, Sp, Sv, Sz); however, Sa was selected as the principal parameter for interpretation because it represents the mean height deviation across the entire scanned surface and is widely accepted in dental surface topography analyses. Parameters such as Sp and Sv are highly sensitive to isolated surface irregularities and may not accurately represent the general characteristics of the surface [36, 37]. To enhance transparency, additional parameters were included as supplementary data.

This study has several limitations that should be considered when interpreting the findings. The blood used for clot formation was obtained from a single donor. Since coagulation parameters and clot ultrastructure may vary between individuals, the generalizability of the blood clot-related findings may be limited. Future studies incorporating blood from multiple donors are warranted to account for biological variability. Therefore, the current study should be considered a preliminary investigation within a controlled in vitro framework. Although this study aimed to standardize in vitro conditions for evaluating surface topography, it does not fully replicate the complex environment of the clinical setting. The use of split root samples enabled high-resolution imaging and quantitative surface analysis, but it may have altered the natural geometry of the canal and affected clot stability. Additionally, the clot was formed under atmospheric conditions with a minimal volume of blood (5 µL) and incubated for a fixed 10 min, which may not reflect in vivo hemodynamic or clot maturation. Future studies should explore more clinically relevant models, including intact teeth with simulated periapical pressures, microfluidic blood delivery systems, and variable clotting times to better mimic physiological conditions in regenerative endodontics. Additionally, the complete removal of EDTA before blood application was not chemically verified in this study. Although a standardized saline rinse was performed after EDTA activation, the possible presence of residual EDTA may have influenced clot structure due to its calcium-chelating effects. Another important limitation of this study is the lack of inclusion of immature teeth, which are more representative of the target population in regenerative endodontic procedures. Although the extracted teeth were obtained from individuals aged 18 to 50 years, age-related variations in dentin structure—such as narrowing of dentinal tubules, increased mineralization, and reduced permeability—may influence the interaction between irrigants and dentin surfaces. As such, the biological differences between mature and immature dentin should be considered when assessing the applicability of these results to clinical regenerative protocols. Moreover, although increased dentin surface roughness has been suggested to facilitate scaffold retention by exposing collagen fibrils and dentinal tubules, the direct relationship between dentin surface roughness and blood clot stability has not been experimentally confirmed and should therefore be considered a biologically plausible hypothesis rather than an established mechanism. Finally, unlike conventional irrigation techniques, the SWEEPS laser modality has demonstrated potential to remove the smear layer even without the adjunctive use of EDTA, owing to its ability to generate shockwave-enhanced cavitation and fluid movement [25, 38]. Although EDTA was used in this study to maintain uniformity across groups, future investigations could isolate this feature to evaluate SWEEPS’ independent efficacy on dentin conditioning.

## Conclusion

Within the limitations of this in vitro study, all final irrigation activation techniques tested were found to significantly increase dentin surface roughness compared to conventional needle irrigation, while producing similar roughness profiles among themselves. These surface alterations may influence the dentin–clot interface; however, the findings should be interpreted with caution, as they were obtained under standardized laboratory conditions. In EDTA-treated groups, the lower surface roughness of the blood clot compared to the control may be associated with the anticoagulant properties of EDTA. Although EDTA activation appears to create dentin surface characteristics that may be favourable for RET, its potential effects on clot architecture highlight the need for careful biological interpretation. Future investigations should incorporate biologically relevant models and evaluate not only surface roughness but also additional surface characteristics such as microhardness, surface energy, and surface chemistry, while considering different canal morphologies and the biological behaviour of blood cells. Further in vivo and clinical studies are required to determine the clinical relevance of these surface alterations and to better understand how irrigation activation protocols influence scaffold formation during regenerative endodontic treatment.

## Data Availability

No datasets were generated or analysed during the current study.
